# Self-Assembled Nanomicellar Formulation of Docetaxel as a Potential Breast Cancer Chemotherapeutic System

**DOI:** 10.3390/life12040485

**Published:** 2022-03-27

**Authors:** Meshal Alshamrani, Navid J. Ayon, Abdullah Alsalhi, Omowumi Akinjole

**Affiliations:** 1 Department of Pharmaceutics, College of Pharmacy, Jazan University, P.O. Box 114, Jazan 45142, Saudi Arabia; alsalhi@jazanu.edu.sa; 2 Proteomics Center of Excellence, Chemistry of Life Processes Institute, Northwestern University, Evanston, IL 60208, USA; navid.ayon@northwestern.edu; 3 Laboratory of Future Nanomedicines and Theoretical Chronopharmaceutics, Division of Pharmaceutical Sciences, School of Pharmacy, University of Missouri-Kansas City, Kansas City, MO 64108, USA; voakff@mail.umkc.edu

**Keywords:** nanomicelles, vitamin E TPGS delivery, enhanced solubility, anticancer activity, breast cancer, docetaxel

## Abstract

Docetaxel (DTX) is classified as a class IV drug that exhibits poor aqueous solubility (6–7 µg/mL in water) and permeability (P-glycoprotein substrate). The main objective of this study was to construct, characterize, and evaluate docetaxel loaded nanomicellar formulation *in vitro* for oral delivery to enhance the absorption and bioavailability of DTX, as well as to circumvent P-gp efflux inhibition. Formulations were prepared with two polymeric surfactants, hydrogenated castor oil-40 (HCO-40) and D-α-Tocopherol polyethylene glycol 1000 succinate (VIT E TPGS) with solvent evaporation technique, and the resulting DTX nanomicellar formulations were characterized by proton nuclear magnetic resonance spectroscopy (1H NMR), Fourier Transform Infrared Spectroscopy (FT–IR), X-ray powder diffraction (XRD), and transmission electron microscopy (TEM). Proton NMR, FT–IR, and XRD data indicated that DTX was completely encapsulated within the hydrophobic core of the nanomicelles in its amorphous state. TEM data revealed a smooth spherical shape of the nanomicellar formulation. The optimized formulation (F-2) possessed a mean diameter of 13.42 nm, a zeta potential of −0.19 mV, with a 99.3% entrapment efficiency. Dilution stability study indicated that nanomicelles were stable up to 100-fold dilution with minimal change in size, poly dispersity index (PDI), and zeta potential. *In vitro* cytotoxicity study revealed higher anticancer activity of DTX nanomicelles at 5 µM compared to the native drug against breast cancer cell line (MCF-7) cells. The LC–MS data confirmed the chemical stability of DTX within the nanomicelles. *In vitro* drug release study demonstrated faster dissolution of DTX from the nanomicelles compared to the naked drug. Our experimental results exhibit that nanomicelles could be a drug delivery system of choice to encapsulate drugs with low aqueous solubility and permeability that can preserve the stability of the active constituents to provide anticancer activity.

## 1. Introduction

Docetaxel (DTX) is classified as a plant alkaloid (taxane-derivative) which is widely used as an antineoplastic drug [1] against many cancers such as solid tumors, acute leukemia, Hodgkin and non-Hodgkin lymphoma, colorectal, breast, stomach, lung, and prostate cancer [2,3,4,5]. DTX exerts its pharmacological effect through binding with microtubules-small units called tubulin [6]. At the G2/M cell cycle stage, the binding suppresses the microtubule depolymerization step, which results in cell cycle arrest, and ultimately leads to cell death [7,8]. The anticancer activity of DTX is reported to be two times greater than that of standard anticancer drugs such as paclitaxel [8].

According to Biopharmaceutics Classification (BCS) System, DTX is considered a class IV drug, characterized by poor aqueous solubility (6–7 µg/mL) and permeability [9]. Low solubility and permeability of DTX result in decreased oral absorption and reduced bioavailability, ultimately, limiting the therapeutic efficacy. To maintain the therapeutic level of DTX in the system, higher doses are required, which cause severe systemic toxicity and develops cancer resistance [10]. It has been reported that approximately 40% of the medications on the market and 90% of the medications under production have low aqueous solubility [11]. Common solubility enhancing agents, such as Tween-80 and Cremophor EL, can enhance the aqueous solubility of hydrophobic drugs; however, such excipients can cause severe adverse effects in humans [12,13,14]. Besides low solubility, DTX is a substrate for efflux pump receptor (P-gp) that leads to diminished bioavailability. P-glycoprotein (P-gp), also known as multidrug resistance protein 1 (MDR1) or ATP-binding cassette subfamily B member 1 (ABCB1), is an ATP-dependent drug efflux pump that is responsible for transporting compounds from the intracellular to extracellular space [15,16]. P-gp activation in tumors diminishes accumulation of chemotherapeutic agents inside the cells and prevents it from reaching minimum effective level finally leading to the development of resistance to many currently available anti-cancer drugs, such as paclitaxel and docetaxel [17].

Thus, P-gp activation and high efflux of the drug from the cell, low permeability, and poor aqueous solubility of DTX limit the therapeutic efficacy of this drug [18]. New generation and novel drug delivery systems, such as liposomes, solid lipid nanoparticles (SLNs), and nanomicelles, have been recently used to improve the therapeutic index and to reduce the adverse effects of DTX [19,20,21]. Increasing the aqueous solubility and ultimately bioavailability are the most critical features of the oral formulation of DTX and other similar hydrophobic drugs [22], which can be achieved by using P-gp inhibitors, self-assembled emulsifiers, and nanocarriers, including nanomicelles [23,24]. Nanomicelles are synthesized from both ionic and nonionic surfactants, and they are developed by the supramolecular assembly of hydrophobic heads and hydrophilic tails, in which a colloidal dispersion is formed [25]. In addition, DTX encapsulation inside the hydrophobic core of the nanomicelles leads to higher stability, solubility, and more permeability [26]. D-α-Tocopherol polyethylene glycol 1000 succinate (VIT E TPGS), an FDA approved safe adjuvant, is a potent P-gp inhibitor; hence, it can help to maintain the therapeutic level of drug inside cancer cells by preventing active efflux [27,28,29]. VIT E TPGS can also withstand high temperature and is stable at pH 4.5–7.5 [30]. Hydrogenated castor oil 40 (HCO-40) is a safe solubility enhancing excipient that can help to solubilize low water soluble drugs [31] and is used to improve gastrointestinal absorption of poorly water soluble drugs which, in turn, increases bioavailability and therapeutic efficacy of the active pharmaceutical ingredient [32]. We used these two amphiphilic polymeric surfactants, hydrogenated castor oil 40 (HCO-40) and Vitamin E TPGS (VIT E TPGS), to prepare DTX nanomicellar formulation to increase the solubility and bioavailability of DTX. The formulations were analyzed by various analytical techniques such as 1H NMR, FT–IR, XRD, LC–MS, TEM, dilution stability study, particle size, zeta potential, PDI, and encapsulation efficiency for characterization. A cytotoxicity study was then performed using the MCF-7 breast cancer cell line to compare the efficacy of the nanomicellar formulation with DTX itself to analyze the advantages of nanomicelles as a potential drug delivery system for anticancer activity. 

## 2. Materials and Methods

### 2.1. General

Docetaxel was obtained from Fischer Scientific with >99% purity. Hydrogenated castor oil 40 (HCO-40) was purchased from the Peboc division of Eastman Company, (Llangefni, UK). Vitamin E TPGS was obtained from Parchem Inc., (New Rochelle, NY, USA). HPLC grade acetonitrile, methanol, and formic acid were procured from Fisher Scientific (Pittsburgh, PA, USA). CellTiter 96^®^ AQueous nonradioactive cell proliferation assay (MTS) kit was obtained from Promega Corp (Madison, WI, USA). MCF-7 cells were procured from the American Type Culture Collection (ATCC, Manassas, VA, USA).

### 2.2. Preparation of DTX Loaded Nanomicelles

**Figure 1:**
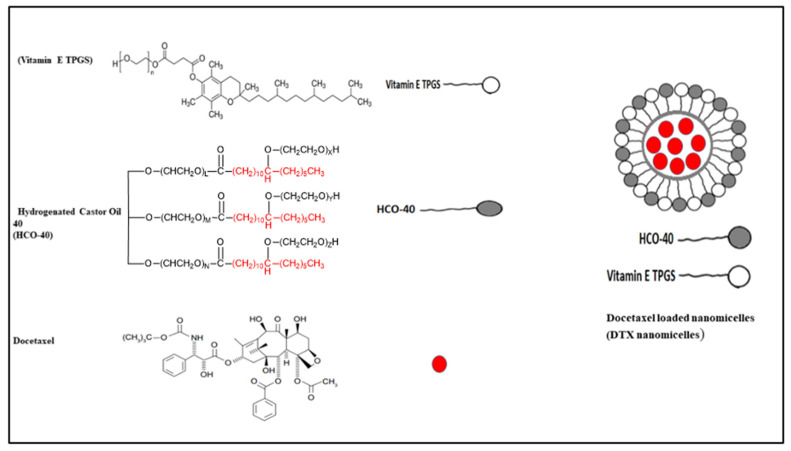
Chemical structure of docetaxel (DTX), HCO-40, VIT E TPGS, and representative cartoon portraying the DTX nanomicelle structure.

Nanomicellar formulations loaded with 0.2% DTX were prepared following the solvent evaporation and film rehydration technique described by Alshamrani et al. and Gote et al. [33,34]. Briefly, a predetermined amount (10 mg) of DTX was accurately weighed and dissolved in ethanol. Then, HCO-40 and VIT E TPGS were weighed out and dissolved separately in ethanol, before being mixed together, to which the DTX ethanolic solution was then added in a dropwise manner. The polymers-drug ratio selection was based on CMC (critical micellar concentration) from previous work [33,34]. Next, the organic phase, ethanol was evaporated using a high-speed vacuum (Genevac, Ipswich, Suffolk, UK) for 8 h to obtain a thin film. The resultant thin film was then rehydrated in deionized (DI) water (5 mL) and vortexed for 5–10 min. Lastly, all the formulations were filtered with a 0.22 µm filter membrane to remove unentrapped DTX aggregates and other foreign particulates. Among four different formulations, F-2 exhibited smaller size, higher entrapment, and loading and, hence, is used for further characterization. The chemical structures of docetaxel, HCO-40 and VIT E TPGS and a representative structure of the nanomicelles are listed in Figure 1.

### 2.3. Characterization of the Synthesized DTX Loaded Nanomicellar Formulations with 1H NMR, FT–IR, and XRD

**Figure 2:**
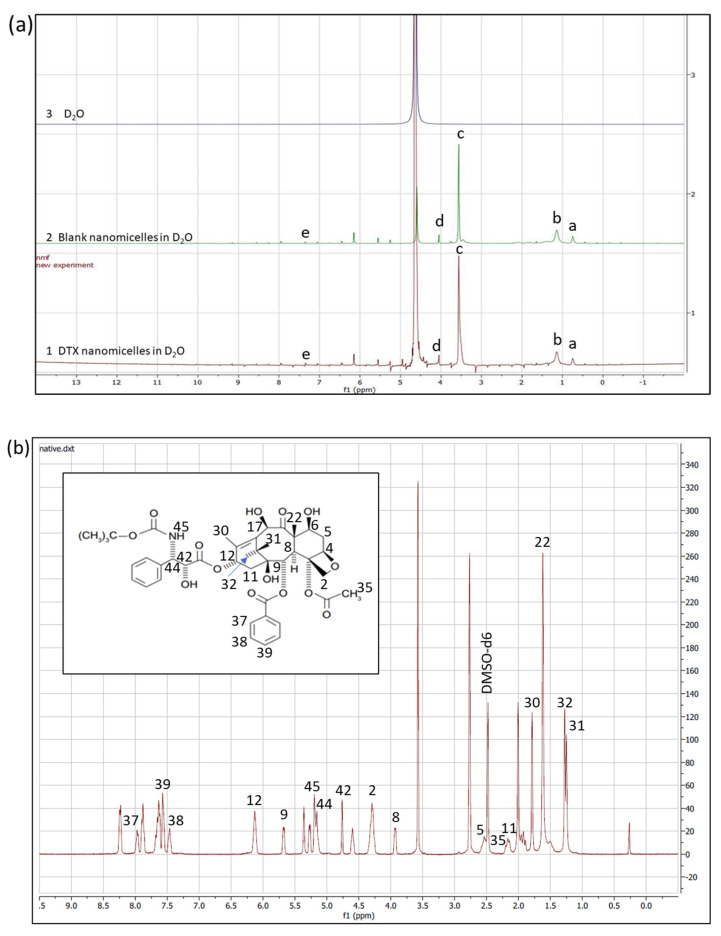
Qualitative 1H NMR analysis of DTX nanomicellar formulation. (**a**) 1H NMR spectrum for (1) DTX nanomicelles in D_2_O, (2) blank nanomicelles in D_2_O, and (3) D_2_O. (a, –CH_3_, δ = 0.7 ppm; b, =CH_2_, δ = 1.3 ppm; c, -CH_2_-CH_2_-O, δ = 3.8 ppm, d, –OH of PEG, δ = 4.2 ppm; e, aromatic protons of VIT E TPGS, δ = 7.3 ppm) (**b**) 1H NMR spectrum of native DTX in DMSO-d6.

**Figure 3:**
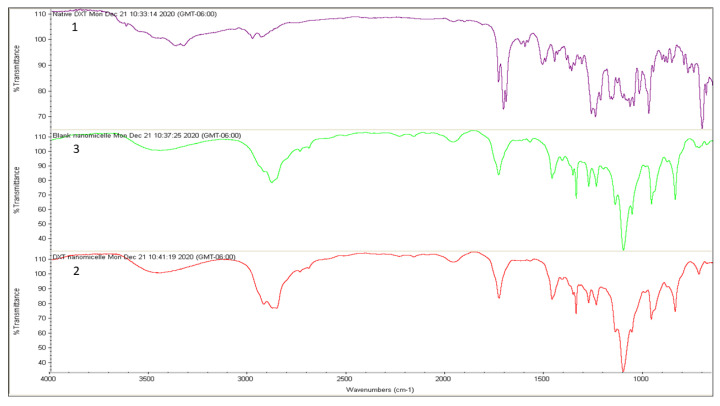
Qualitative FT–IR analysis of DTX nanomicelle. FT–IR spectrum for native (**1**) native DTX, (**2**) DTX nanomicelles, and (**3**) blank nanomicelles.

**Figure 4:**
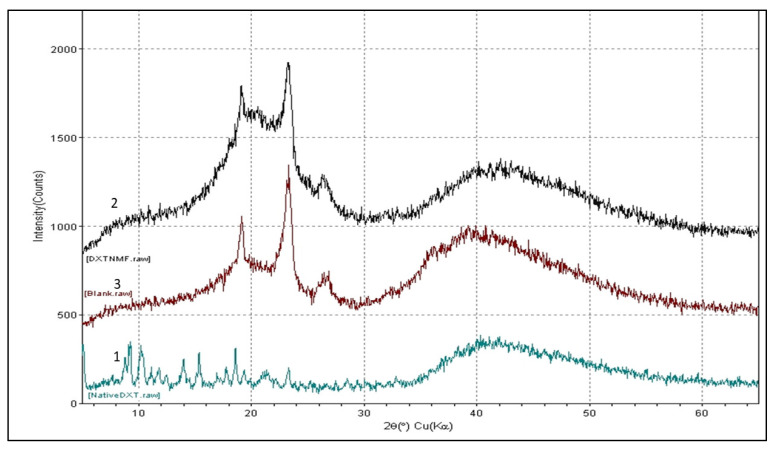
Qualitative XRD analysis of DTX nanomicelle. XRD spectrum for native (**1**) native DTX, (**2**) DTX nanomicelles, and (**3**) blank nanomicelles.

To perform the 1H NMR experiment, the freeze-dried powder of DTX nanomicellar formulation (F-2) and blank nanomicellar formulation were dissolved in D_2_O at a concentration of 2 mg/mL. The 1H NMR spectra was recorded on Varian Inova 400 MHz NMR spectrometer (Varian, Palo Alto, CA, USA) at 25 °C. Chemical shifts were measured in parts per million (0–12 ppm) with a delay period of 4 s. For easy comparison, spectra from all three samples including solvent blank (D_2_O) are plotted in one graph (Figure 2a). FT–IR spectra was acquired on Thermo-Scientific Nicolet iZ10, with an ATR diamond and DTGS detector, using pure DTX, blank, and DTX loaded nanomicelles (F-2) at a scanning range of 650–4000 cm^−1^ (Figure 3). XRD analysis was carried out to determine the crystallinity of the formulation components of the DTX alone, DTX nanomicelles (F-2), and blank nanomicelles (Figure 4). The MiniFlex-automated X-ray diffractometer (Rigaku, The Woodlands, TX, USA) was used which is equipped with Ni-filtered Cu Kα radiation operating at 30 kV and 15 mA at room temperature. The diffraction angle covered was from 2θ = 5° to 2θ = 40° with a step size of 0.05°/step and a counting time of 2.5 s/steps (1.2°/min) for 30 min. The diffraction patterns were processed using Jade 8+ software (Materials Data, Inc., Livermore, CA, USA).

### 2.4. Size, PDI, and Zeta Potential

Size, PDI, and zeta potential of DTX nanomicelles were acquired on Zeta Sizer (Zetasizer 3600 Nano ZS, Malvern Instruments Ltd., Worcestershire, UK), which employs a dynamic light scattering technique for particle size measurement at a detection angle of 90° at 25 °C [35]. Briefly, 1 mL of DTX nanomicellar formulation (F-2) (1 mg/mL) was placed into a glass cuvette. The sample was measured at a scattering angle of 173° and 25 °C. Data were collected in triplicate measurements and expressed as mean ± standard deviation (SD) using Zetasizer software version 6.01 (Worcestershire, UK). The morphology of the nanomicelles was analyzed by Transmission Electron Microscopy (TEM). Briefly, 50 µL of DXT nanomicelles (F-2) was placed on a carbon-coated copper grid. Then, phosphotungstic acid was used to stain the samples for data acquisition.

### 2.5. Entrapment Efficiency and Drug Loading

The total amount of DTX entrapped inside the core of the nanomicelles was determined by reversed-phase HPLC (RP–HPLC) with a Shimadzu LC pump (Columbia, MA, USA), Alcott autosampler (model 718 AL), Shimadzu UV/Vis detector (SPD-20AV), and Phenomenex C18 column (Spherisorb 250 × 4.60 mm, 5 μm). An isocratic flow of the mobile phase composed of acetonitrile, water and formic acid (80%/19.9%/0.1%, *v*/*v/v*) was used at 0.5 mL/min flow rate. Detection was performed with a UV detector at 231 nm. Standard docetaxel solution was prepared at 1 mg/mL with the mobile phase, serially diluted in the mobile phase, of which 30 µL was injected to collect data. Area under the curve for the serially diluted docetaxel solution was then used to construct the calibration curve, which showed the highest linearity with R^2^ = 0.9998 for a range of 1 to 100 µg/mL. To analyze the amount of docetaxel entrapped inside the nanomicellar formulations, 1 mL of each formulation was centrifuged at 10,000 rpm at 4 °C for 10 min, and 500 µL of the supernatant was collected and transferred to a new vial followed by lyophilization to obtain a thin film. Next, 500 µL of dichloromethane was added to reverse the micelle and release all the DTX in the surrounding organic phase. The solution mixture was evaporated under high–speed vacuum to obtain a solid pellet of reversed micelles which was then reconstituted with mobile phase and the sample was then injected into the HPLC. The amount of docetaxel trapped inside the nanomicelles (entrapment efficiency) and the amount of docetaxel entrapped compared to the added amount (loading efficiency) were calculated according to the following equations and summarized in Table 1, where the total amount of docetaxel used in each formulation was 10 mg.

(1) $$\mathrm{Entrapment}\;\mathrm{efficiency}=\frac{\mathrm{amount}\;\mathrm{of}\;\mathrm{DTX}\;\mathrm{quantified}\;\mathrm{in}\;\mathrm{nanomicelles}}{\mathrm{amount}\;\mathrm{of}\;\mathrm{DTX}\;\mathrm{added}\;\mathrm{in}\;\mathrm{the}\;\mathrm{nanomicelles}}\;\times 100$$

(2) $$\mathrm{Loading}\;\mathrm{efficiency}=\frac{\mathrm{amount}\;\mathrm{of}\;\mathrm{DTX}\;\mathrm{quantified}\;\mathrm{in}\;\mathrm{nanomicelles}}{\mathrm{amount}\;\mathrm{of}\;\mathrm{DTX}\;\mathrm{added}+\mathrm{amount}\;\mathrm{of}\;\mathrm{polymers}\;\mathrm{used}}\;\times 100$$

### 2.6. In Vitro Release of Docetaxel from the DTX Nanomicelles

RP-HPLC method was used to analyze the *in vitro* release of docetaxel from DTX nanomicelles as described above. Briefly, dialysis was performed by adding 1 mL of DTX nanomicelles to a dialysis bag with a molecular weight cut off (MWCO) of 2 kDa which was then immersed in a 15 mL centrifuge tube containing 5 mL PBS (pH 7.4). To increase the solubility of the released DTX from the nanomicelles, 0.1% of Tween–20 was added to the PBS buffer. A 1.0 mL sample was taken every 24 h and replaced with an equivalent volume of the new PBS solution to maintain sink condition. The concentration of DTX released from nanomicelles in PBS buffer solution was calculated using the standard curved determined by the RP–HPLC method stated above.

### 2.7. Dilution Stability of DTX Nanomicellar Formulation

DLS (Zetasizer Nano ZS, Malvern Zetasizer, Westborough, MA, USA) was used to investigate the influence of dilution on hydrodynamic size, zeta potential, and PDI. Using DI water, 0.2% DTX nanomicelles were diluted up to 100 times and the particle size was measured.

### 2.8. In Vitro Cytotoxicity: MTS Assay

Cytotoxicity of blank and DTX loaded nanomicelles was evaluated by MTS using [3-(4,5-dimethylthiazol-2-yl)-5-(3-carboxyMethoxyphenyl)-2-(4-sulfophenyl)-2H Tetrazolium Solution] assay on human breast cancer cell line (MCF-7) according to the manufacturer’s protocol. MCF-7 cells were cultured first in T75 flasks in Dulbecco’s Modified Eagle Medium (DMEM) that contains high l-glutamine and glucose concentrations, 1% nonessential amino acids, and 10% FBS (heat-inactivated). To the cells in the DMEM media were then added 100 IU/mL streptomycin and 100 IU/ mL penicillin to protect cells from bacteria. The pH of the medium was adjusted to 7.4, similar to human blood pH. Cells were incubated in an atmosphere containing 5% CO_2_ and 90% relative humidity at 37 °C. Until cells reached 90% confluence, the media was replaced every alternate day. This cellular suspension was then used to dispense ~1 × 10^4^ cells in 100 µL of DMEM media containing 10% FBS in each well of a 96-well plate. Cells were cultured and allowed to attach for 24 h at 37 °C in a 5% CO_2_ environment. All formulations were prepared in serum-free DMEM media and filtered with a sterile 0.22 µm filter membrane under laminar flow inside a biosafety hood. DMEM media was then removed and cells were rinsed with DPBS twice.

Each well containing cells were then exposed to different concentrations of docetaxel nanomicellar formulation, 5, 10, 50, and 100 µM, and blank nanomicelles (DPBS) for 24 h at 37 °C. Serum-free media and 10% Triton X-100 served as negative and positive controls, respectively. After 24 h of incubation, an MTS stock solution of 5 mg/mL was added to all the wells. Cell viability percentage was calculated based on the absorbance value of sample, negative and positive control at 450 nm where the amount of formazan product is directly proportional to the number of viable cells. Therefore, cell viability was expressed according to the following equation:

Cell viability (%) = (Abs of sample − Abs of serum-free media)/(Abs of Triton X 10% − Abs of serum-free media) ∗ 100(3)

### 2.9. Liquid Chromatography (LC)–Tandem Mass Spectrometry (LC–MS/MS) 

LC-MS/MS analysis was carried out to ensure that DTX remains chemically stable inside the hydrophobic core of the nanomicelles. In brief, the DTX nanomicelles formulation (10 µM DTX) was dissolved in a solvent containing water, acetonitrile, and formic acid, (49.95%/49.95%/0.1%, *v*/*v*/*v*). The control (10 µM of native DTX) was dissolved in an aliquot of the same organic solvent spiked with blank nanomicelles to account for the interaction of polymers with the drug and hence its effect on the stability of drug. LC–MS/MS analysis was performed using a method we previously developed to analyze docetaxel in solid-dispersion dosage form [36]. The LC–MS/MS analysis was carried out on an AB Sciex 3200 QTrap mass spectrometer (Foster City, CA, USA) coupled to a Shimadzu UFLC system (Columbia, MD, USA) using electrospray ionization (ESI) and run with Analyst version 1.4.2 software. Data analysis and quantification were performed using the automated quantitative optimization routine in Analyst version 1.6 (Redwood, City, CA, USA). The separation was achieved with a C8 column (125 mm × 3 mm, 5 µm) at a flow rate of 0.3 mL/min with the following gradient of mobile phase A (water with 0.1% formic acid) and B (acetonitrile with 0.1% formic acid): 10% B for 0-4 min, 10–100% B for 4–10 min with a 0.5 min ramp down to 10% B and a 4 min equilibration at initial conditions post acquisition for next injection. The MS conditions, and the optimal parameters for the selected precursor/product ion pairs for DTX, are shown in Table 2.

### 2.10. Statistical Analysis

At least three triplicates for each sample type were used in all experiments that were performed, and results were expressed as mean ± SD. In addition, student’s t-test or one-way ANOVA was employed to determine the statistical significance among groups where *p* < 0.05 indicated statistical significance in relevant experiments. 

## 3. Results and Discussion

### 3.1. Characterization of the Synthesized Nanomicelles Loaded with DTX with 1H NMR, FT–IR, and XRD

Proton NMR spectra of HCO-40 and VIT E TPGS, two amphiphilic surfactant polymers, showed intense peaks for oxyethylene group, -CH_2_-CH_2_-O (δ = 3.8 ppm, c) and modest signals for methylene group, =CH_2_ (δ = 1.3 ppm, b), and methyl group, –CH_3_ (δ = 0.7 ppm, a) (Figure 2a). Weak signal for hydroxylic protons of PEG (δ = 4.2 ppm, d) and aromatic protons of VIT E TPGS (δ = 7.3 ppm, e) were also observed. Extremely weak signal for the hydroxylic and the aromatic protons are due to significantly outnumbered alkyl protons present in both the VIT E TPGS [24] and HCO-40 [37], which also aligns with published literature [38,39,40,41]. Comparison to spectra for blank nanomicelles and DTX nanomicelles in D_2_O revealed these similar set of peaks confirming the presence of the stable polymers in the nanomicelles. However, none of the major peaks for the protons of docetaxel, as denoted in Figure 2b, were observed in DTX nanomicellar formulation (F-2) [42,43]. The absence of these majority of the peaks for protons of various functional groups of DTX in the nanomicellar formulation indicated complete entrapment of docetaxel inside the hydrophobic core of the nanomicelles.

FT–IR analysis of DTX nanomicelles, blank nanomicelles, and DTX alone was performed to investigate docetaxel entrapment inside the core of the nanomicelles. Figure 3 depicts that spectra of DTX alone that aligns with previously published data [19]. Spectral comparison of blank nanomicelles and DTX nanomicelles solutions revealed a similar set of peaks. It can be noted that the majority of the peaks for DTX were not observed in DTX nanomicelles (Figure 3), confirming that DTX was effectively and wholly entrapped inside the hydrophobic core of the nanomicelles.

XRD data revealed the presence of a crystalline form of DTX with its characteristic diffraction peaks at 7.3, 8.8, 13.7, 17.2, and 20.2 ± 0.2° two-theta [44] (Figure 4). On the contrary, DTX nanomicelles and blank nanomicelles have a nearly superimposed graph with a characteristic peak at 19.2° and 22.4°, which can be attributed to the polymers (HCO-40 and VIT E TPGS) with the absence of characteristic peaks of DTX. Thus, the XRD data also indicated that DTX was entrapped effectively inside the nanomicelles in its amorphous form. Collectively, 1H NMR, FT–IR, and XRD data (Figure 2, Figure 3 and Figure 4) revealed intermolecular interactions between docetaxel and the polymers (HCO-40 and VIT E TPGS), along with a complete entrapment of docetaxel inside the nanomicelle core and disappearance of docetaxel’s crystallinity.

### 3.2. Size, PDI, Zeta Potential, and Surface Morphology

**Figure 5:**
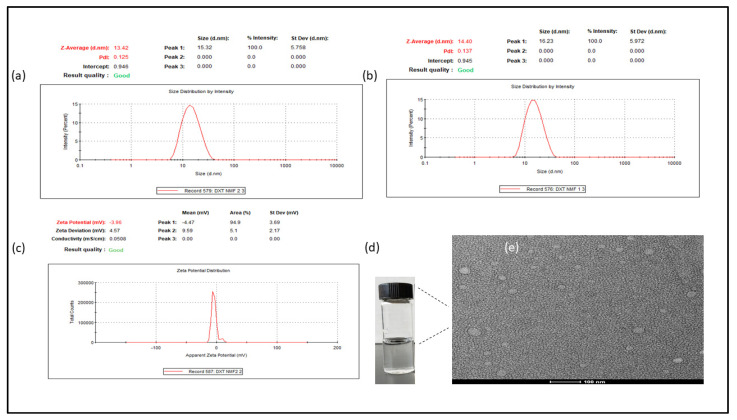
Size distribution of (**a**) DTX nanomicelles (**b**) blank nanomicelles, (**c**) zeta potential of DTX nanomicelles, (**d**) visual inspection of DTX nanomicelles, and (**e**) TEM image of DTX nanomicelles showing round smooth morphology.

Amphiphilic polymeric surfactants, HCO-40 and VIT E TPGS, form spherical nanomicelles above the critical micellar concentration, in which hydrophobic core remains inside the micelles whereas the external layer is made up of hydrophilic groups. Hence, nanomicelles synthesized with amphiphilic polymers can entrap lipophilic compounds in their cores and act as an efficient drug delivery system for them, enhancing their solubility dramatically. The DTX nanomicellar formulations prepared (F1–F4) had a hydrodynamic size range of 13.42–28.23 nm (Figure 5A), PDI of 0.10–0.22 (Figure 5B), and zeta potential of (−0.912) to (−4.896) mV (Figure 5C). The optimized formulation F-2 (Table 1) had the lowest size, PDI, and maximum entrapment and loading efficiency. The entrapment efficiency of around 99% indicate near to complete entrapment of the hydrophobic drug DTX within the core of the nanomicelles. In addition, among all the nanomicellar formulations, the smallest particle size of F-2 could indicate a more critical hydrophobic-hydrophobic interaction between polymers and drugs. It is noteworthy that comparison between the blank nanomicelles and DTX loaded nanomicelles, in terms of size, revealed smaller size for the latter, probably due to the nature of the hydrophobic-hydrophobic interactions between the DTX and the polymers which results in shrinkage of the nanomicelles’ core. It is important to note that the aqueous solubility of DTX increased from 6 µg/mL (0.006 mg/mL) up to 2000 µg/mL (2 mg/mL), representing about 333-fold increase in the nanomicellar formulation compared to the standard drug alone. While visual inspection of DTX nanomicelles revealed a clear solution (Figure 5d), TEM showed a smooth spherical shape (Figure 5e).

### 3.3. Entrapment Efficiency and Drug Loading

Varying ratio of HCO-40 and VIT E TPGS led to varied entrapment efficiency ranging from 18.32% to 99.30%, as well as varied loading efficiency ranged from 1.12% to 3.62% in F1–F4 (Table 1). The amount of DTX was constant among all four formulations (2 mg/mL) and it can be noted that the formulations exhibited varying loading and entrapment efficiencies due to the difference in the polymer concentration. Among formulations F1–F4, F-2 exhibited both the highest entrapment efficiency, and the highest loading efficiency. F-1 showed precipitation of DTX due to low concentrations of both polymers which were 0.1% (wt %) for both VIT E TPGS and HCO-40. The greater entrapment and loading efficiency seen in F-2 is probably due to considerable hydrophobic–hydrophobic interactions between the hydrophobic drug and the hydrophobic core of the nanomicelles. From our experiments we also observed that in general higher amount of HCO-40 among the two polymers used yielded smaller sized nanomicelles with better entrapment efficiency and drug loading.

### 3.4. Dilution Stability of DTX Nanomicellar Formulation

**Figure 6:**
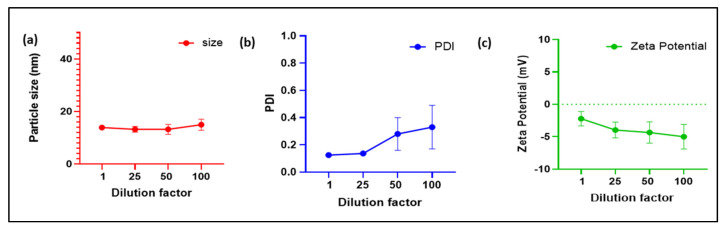
Dilution stability study of the DTX nanomicelles characterized by (**a**) particle size, (**b**) PDI, and (**c**) zeta potential at room temperature.

Dilution stability was assessed after diluting the nanomicellar formulation up to 100 times of their original volume, and by measuring the size, PDI, and zeta potential of DTX nanomicelles (Figure 6). The size of the nanomicelles increased from 13.36 nm to 14.99 nm after 100 times dilution, the PDI increased from 0.125 to 0.330, and the zeta potential reduced from −2.21 mV to −4.99 mV. According to these findings, dilution up to 100 times did not influence nanomicellar size and caused considerable PDI and zeta potential changes. Comparable results were reported in prior studies with identical polymers to improve delivery of different ocular medications such as triamcinolone acetonide, curcumin, and tacrolimus [33,34,45].

### 3.5. In Vitro Release of Docetaxel from the Nanomicellar Formulation

**Figure 7:**
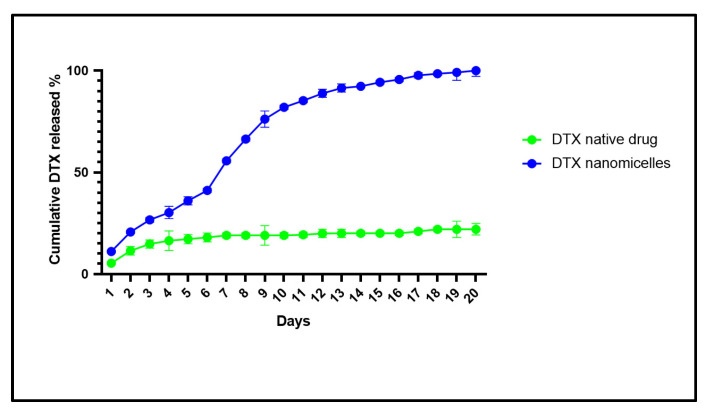
*In vitro* drug release of DTX from nanomicelles vs. DTX native drug evaluated in PBST solution.

*In vitro* drug release study is essential to determine the time required for a formulation to release the active ingredient. Analyses were performed with very diluted samples, so the DTX release is not limited by its solubility. *In vitro* drug release study was performed to emulate the pharmacokinetic pattern of drug release and mimic the performance of the nanomicelles as a delivery system in vivo in PBS + 1% tween-20 (PBST) to simulate similar features of in vivo conditions such as the physiological pH. Sink condition was maintained by replacing withdrawn volume with constant shaking at 37 °C. The release study of the DTX nanomicelles was conducted in triplicates for samples at each time point. As evident from Figure 7, DTX was released from DTX nanomicelles in a controlled and sustained manner, with around 99% drug released for over more than 20 days. This enhanced release of docetaxel from the nanomicellar formulation is due to (a) reduced average particle size (∼13 nm) of DTX nanomicelles, and (b) surfactants (VIT E TPGS and HCO-40) reducing the surface tension between DTX and the external aqueous layers. However, naked DTX, being very hydrophobic, showed a poor dissolution in the aqueous media, which can be seen from the nearly unchanged amount of docetaxel being released and remain soluble over the period of 20 days. This study suggests that DTX nanomicelles can sustain the release of DTX when administered orally as nanomicelles.

### 3.6. In Vitro Cell Viability Assay against MCF-7 Cell Line

**Figure 8:**
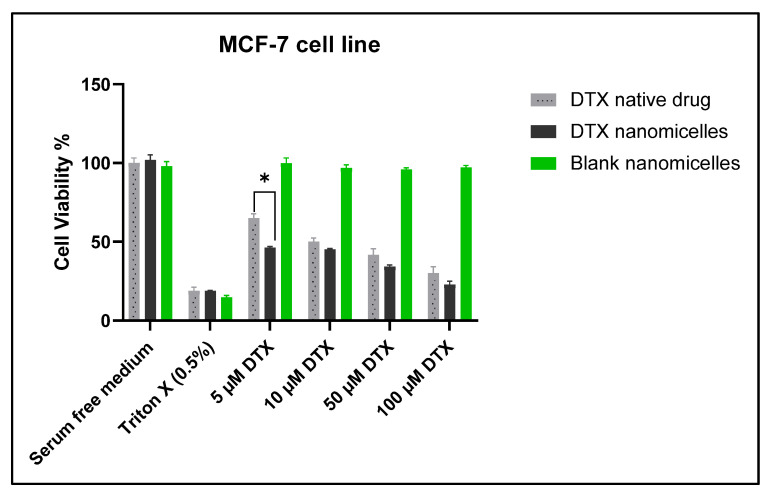
Cell viability assay (MTS) on MCF-7 cells after 24 h of exposure to different concentrations of native DTX, DTX nanomicelles, and blank nanomicelles. (* *p* ≤ 0.05 as compared to the corresponding control group, here DTX).

Cell proliferation assay, MTS, was used to test cell viability after 24 h of exposure to blank nanomicelles and nanomicelles loaded with DTX on breast cancer cell line (MCF-7 cells). MTS data revealed that at 5 µM concentration, DTX nanomicelles exhibited higher cytotoxic activity against MCF-7 cell line, with a cell viability of nearly 46%, whereas 65% cell viability was observed with DTX alone (Figure 8). In addition, VIT E TPGS can inhibit P-glycoprotein and multidrug resistance (MDR) mechanisms which in turn can cause accumulation of DTX in the cancer cells. The significant difference can be attributed to (i) higher solubility of docetaxel in nanomicelles (2 mg/mL) compared to docetaxel alone (<7 µg/mL), which is a 333 fold increase in aqueous solubility; (ii) inhibitory effect of VIT E TPGS on P-gp efflux pump that increases the accumulation of DTX in MCF-7 cells by reducing efflux of DTX from intracellular to extracellular space; and (iii) smaller size of nanomicelles (less than 14 nm), which allow them to enter cancer cells very quickly through a different mechanisms such as the enhanced permeability and retention (EPR) [46], passive diffusion, and clathrin and caveolae independent pathways [47]. However, there were no significant differences at 10, 50, and 100 µM concentrations of both naked DTX and DTX nanomicelles (Figure 8) which could probably be due to the saturation of the P-gp transporter activity by high concentration of DTX nanomicelles and DTX alone [48]. It is noteworthy that blank nanomicelles, which had no drug, even at a higher concentration (100 µM), did not show any cytotoxic activity confirming the safety of the polymeric surfactants used in our formulations. Recent studies suggest that the co-administration of VIT E TPGS with chemotherapeutic agents may overcome drug resistance by decreasing drug efflux [28,49,50].

### 3.7. LC–MS/MS Analysis for Chemical Stability

**Figure 9:**
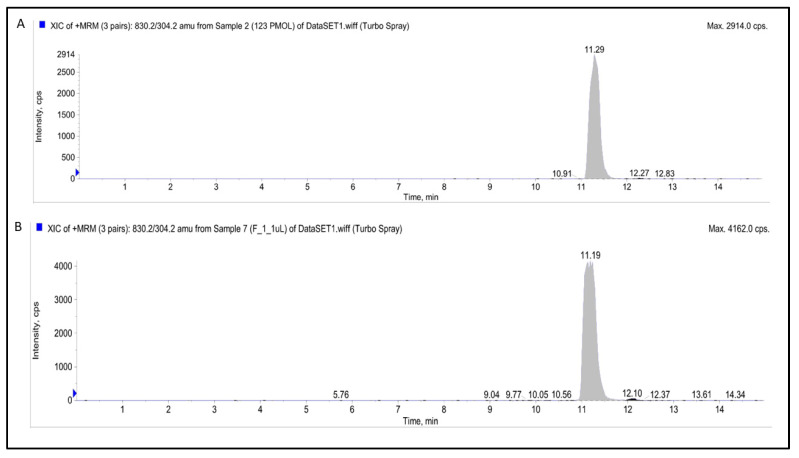
Multiple reaction monitoring (MRM) chromatograms of (**A**) native docetaxel (**B**) docetaxel loaded nanomicelles.

LC–MS/MS analysis of (a) native docetaxel (11.29 min) and (b) docetaxel-loaded nanomicelles (11.19 min) exhibited similar retention time (difference = 0.10 min), m/z, and fragmentation profile (Figure 9), which indicates that the nanomicelles, as a delivery system, maintain the chemical stability of DTX. The result is, indeed, expected because docetaxel is stable inside the hydrophobic core of the nanomicelles which upon release when detected by LC–MS/MS had the same retention time, m/z and fragmentation pattern which is consistent with the data we have published before on docetaxel stability in solid dispersion formulation [36].

## 4. Conclusions

Our study showed successful entrapment of docetaxel inside polymeric nanomicelles made with two FDA-approved polymeric surfactants, namely HCO-40 and VIT E TPGS, with a solvent-evaporation thin-film rehydration technique that demonstrated 333-fold increase in aqueous solubility as well as better anticancer activity against MCF-7 cell line. Such solubility enhancement is expected to enhance oral absorption of drug leading to better bioavailability and diminish the requirement of higher doses to achieve minimum effective concentration for pharmacological activity; hence, potentially will have lesser side effects. Additionally, our nanomicellar formulation exhibited sustained release of docetaxel that can help reduce dosing frequency to achieve therapeutic efficacy in cancer treatment. The entrapment, stability, and morphology of docetaxel were analyzed and confirmed by various analytical techniques, namely, 1H NMR, FT–IR, XRD, LCMS, and TEM. We believe this study supports and aligns with ongoing research activities in the field of nanomicelles delivery system as a drug delivery vehicle for poorly water-soluble drugs specially hydrophobic cancer therapeutics and can help design in vivo studies for further validation of such approach in cancer treatment and management.

## Figures and Tables

**Table 1:** Size, entrapment, and loading efficiency of DTX nanomicellar formulations at varying weight % ratio of polymers.

Formulation	DTX (wt %)	Polymer Ratio (wt %) (HCO-40: VIT E TPGS)	Size (nm)	Entrapment Efficiency %	Drug Loading %
* F-1	0.2	0.1:0.1	24.23 ± 2.20	18.32 ± 2.32	1.12 ± 0.03
F-2	0.2	2.5:1	13.42 ± 0.62	99.30 ± 1.96	3.62 ± 0.11
F-3	0.2	0.5:0.01	28.23 ± 0.92	35.73 ± 2.23	2.10 ± 0.23
F-4	0.2	2.5:0.01	14.40 ± 0.52	90.12 ± 1.92	3.53 ± 0.21

* Due to low concentrations of polymers in F-1, a white precipitate was observed in the system.

**Table 2:** Mass spectrometry parameters for the most intense MS/MS transition of Docetaxel in positive mode detection with electrospray ionization ^a^.

	Parent Ion (Q1)	Fragment Ion (Q3)	Collision Energy (V)
Docetaxel	830.26	304.2	33

^a^ Global method parameters were: source temperature 300 °C, curtain gas (CUR) 20, ion spray voltage (IS) -4500 V, Gas flow (GS1 and GS2) 50 (arbitrary units), and collision associated dissociation (CAD) High.

## Data Availability

The data presented in this study are available on request from the corresponding author.
